# Supplementary material for: Genotypic Variation in Nickel Accumulation and Translocation and Its Relationships with Silicon, Phosphorus, Iron, and Manganese among 72 Major Rice Cultivars from Jiangsu Province, China
Source: Int J Environ Res Public Health. 2019 Sep 6;16(18):3281. doi: 10.3390/ijerph16183281 (PMC6765936; doi:10.3390/ijerph16183281)
Supplement: Supplementary file 1 [file ijerph-16-03281-s001.pdf]

Table S1. Pearson correlation coefficients between accumulation and translocation of multi-element (Si, Ni, P, Fe, and Mn) in 20 lowest Ni-accumulating rice cultivars.

		Shoot concentrations					Root concentrations					TFs				
		Si	Ni	P	Fe	Mn	Si	Ni	P	Fe	Mn	Si	Ni	P	Fe	Mn
Shoot concentrations	Si	1	0.345	0.006	0.357	-0.175	0.418	-0.247	0.066	0.171	0.445*	-0.216	0.456*	-0.076	-0.238	-0.375
	Ni		1	0.307	0.305	-0.251	-0.181	-0.161	0.007	-0.256	0.239	0.280	0.559*	0.402	0.144	-0.253
	P			1	0.141	0.242	0.142	-0.154	0.553*	0.246	0.000	-0.237	0.182	0.576**	-0.285	-0.011
	Fe				1	-0.215	-0.440	0.056	-0.345	-0.410	-0.263	0.507*	0.147	0.444*	0.539*	0.226
	Mn					1	0.242	-0.389	0.248	0.436	0.079	-0.088	0.136	0.087	-0.359	0.312
Root concentrations	Si						1	-0.152	0.707**	0.845**	0.622**	-0.894**	-0.037	-0.534*	-0.848**	-0.369
	Ni							1	-0.212	-0.292	-0.174	0.027	-0.843**	0.032	0.494*	0.243
	P								1	0.649**	0.398	-0.710**	0.048	-0.346	-0.684**	-0.255
	Fe									1	0.441	-0.802**	0.018	-0.365	-0.916**	-0.273
	Mn										1	-0.323	0.122	-0.352	-0.464*	-0.739**
TFs	Si											1	0.146	0.462*	0.833**	0.271
	Ni												1	0.181	-0.247	-0.232
	P													1	0.355	0.238
	Fe														1	0.407
	Mn															1

Note: The translocation factor of Si, Ni, P, Fe, and Mn in rice was calculated as Cshoot/Croot. * Correlation is significant at the 0.05 level (two-tailed). ** Correlation is significant at the 0.01 level (two-tailed).

Table S2. Pearson correlation coefficients between accumulation and translocation of multi-element (Si, Ni, P, Fe, and Mn) in 20 highest Ni-accumulating rice cultivars.

		Shoot concentrations					Root concentrations					TFs				
		Si	Ni	P	Fe	Mn	Si	Ni	P	Fe	Mn	Si	Ni	P	Fe	Mn
Shoot concentrations	Si	1	-0.227	0.189	0.759**	-0.135	0.789**	-0.619**	0.450*	0.645**	0.634**	-0.460*	0.570**	-0.669**	-0.395	-0.742**
	Ni		1	0.071	-0.205	0.396	-0.310	0.364	-0.067	-0.181	-0.220	0.257	-0.111	0.360	0.243	0.454*
	P			1	-0.010	0.470*	0.122	-0.084	0.899**	0.165	0.162	-0.020	0.071	-0.186	-0.188	0.041
	Fe				1	-0.469*	0.357	-0.541*	0.102	0.176	0.192	-0.078	0.517*	-0.312	0.106	-0.514*
	Mn					1	0.041	0.316	0.503*	0.260	0.252	0.030	-0.290	-0.216	-0.303	0.383
Root concentrations	Si						1	-0.515*	0.417	0.830**	0.614**	-0.829**	0.423	-0.699**	-0.761**	-0.602**
	Ni							1	-0.152	-0.350	-0.157	0.342	-0.946**	0.323	0.299	0.459*
	P								1	0.465*	0.492*	-0.193	0.081	-0.581**	-0.413	-0.230
	Fe									1	0.577**	-0.656**	0.237	-0.739**	-0.902**	-0.446*
	Mn										1	-0.287	0.063	-0.709**	-0.391	-0.716**
TFs	Si											1	-0.307	0.390	0.777**	0.358
	Ni												1	-0.157	-0.179	-0.366
	P													1	0.611**	0.585**
	Fe														1	0.272
	Mn															1

Note: The translocation factor of Si, Ni, P, Fe, and Mn in rice was calculated as Cshoot/Croot. * Correlation is significant at the 0.05 level (two-tailed). ** Correlation is significant at the 0.01 level (two-tailed).

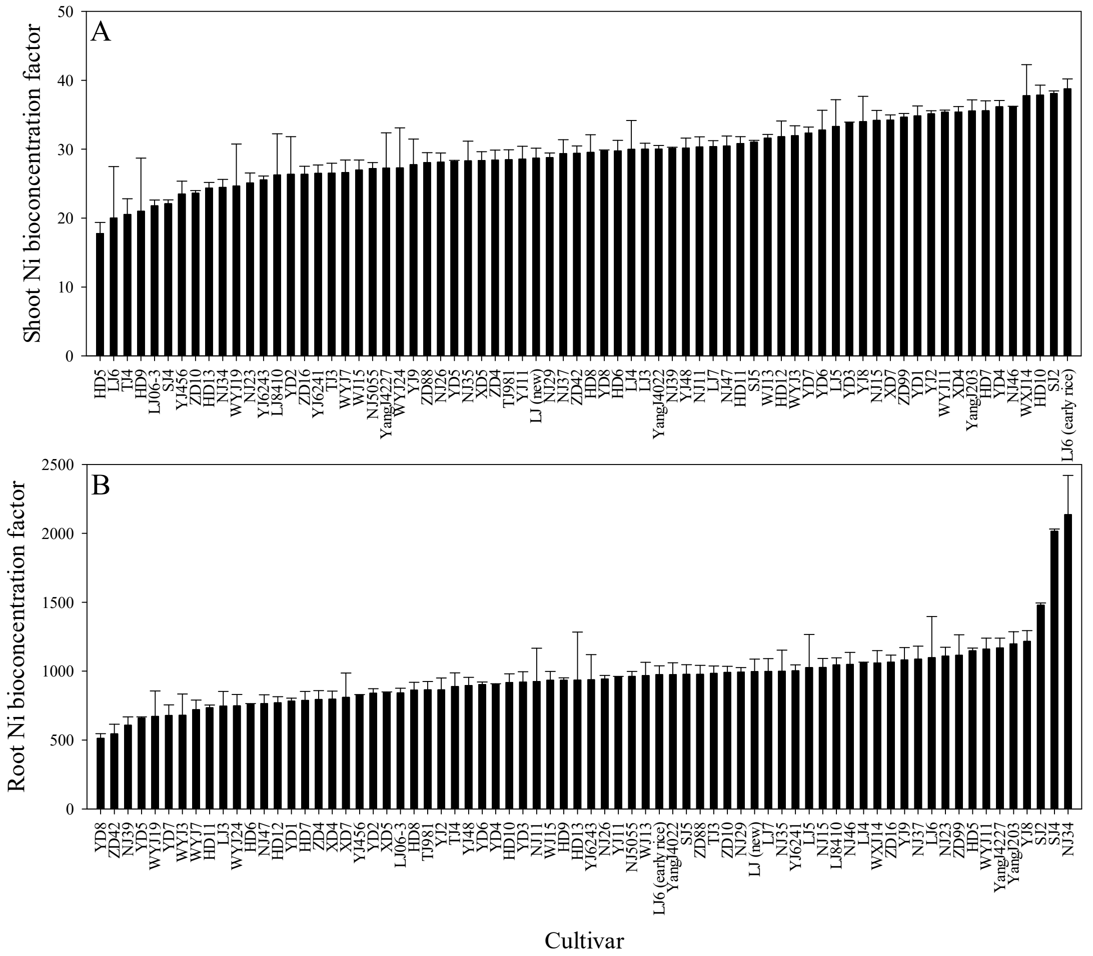

Figure S1. Nickel (Ni) bioconcentration factors in the rice shoots (A) and roots (B). The bioconcentration factor (BCF) of Ni from culture medium to shoots or roots were calculated as: BCF= Cshoots Ni or roots Ni / CNi in culture medium. Data are means ± standard deviation (n=3).

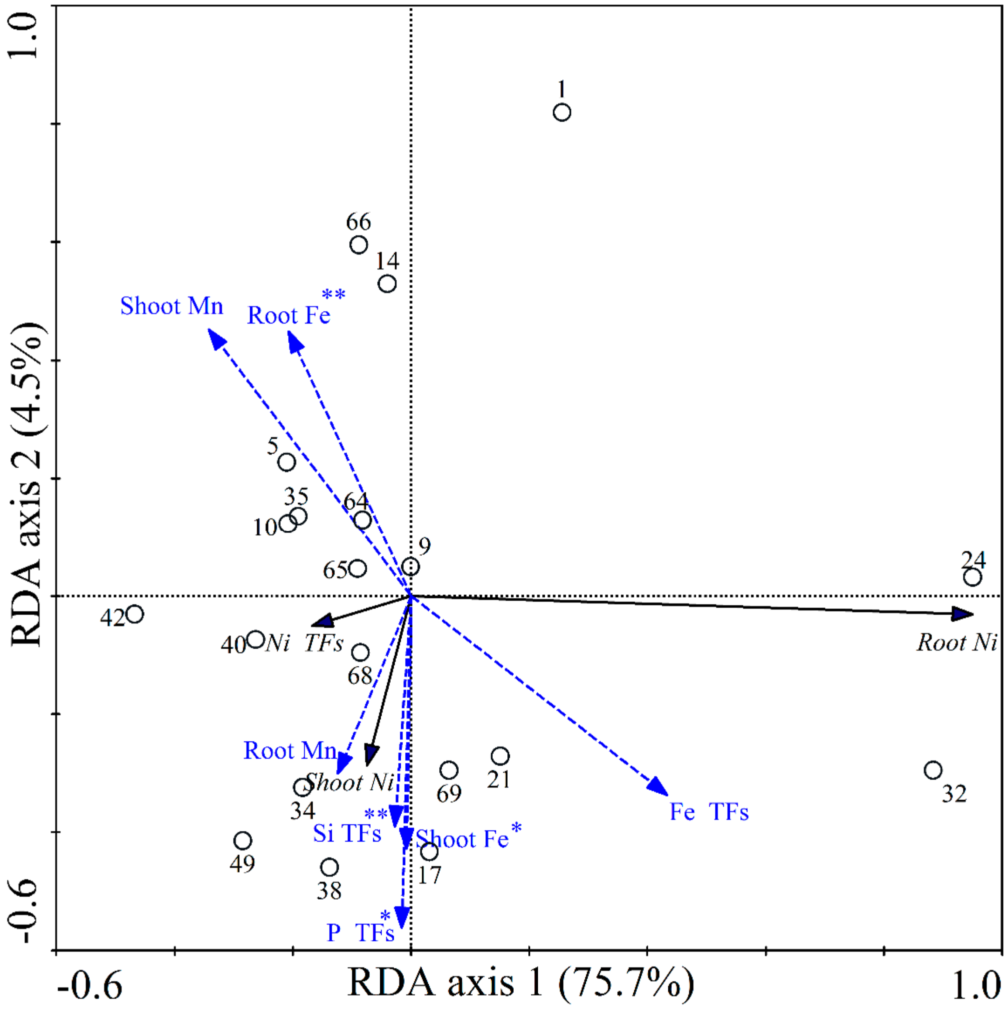

Figure S2. RDA ordination diagrams of the relationships between accumulation and translocation of Ni and multi-element (Si, P, Fe, and Mn) in 20 lowest Ni-accumulating rice cultivars. Shoot Ni, root Ni, and Ni TFs were displayed as black lines with arrows. Shoot Fe, root Fe, root Mn, Si TFs, and Fe TFs were represented by blue lines with arrows. The numbers around the circles "○" represent associated rice cultivars listed in Table 1. *($p < 0.05$) and **($p < 0.01$) represent significant factors influencing Ni accumulation and translocation based on Monte Carlo analysis (the number of permutations = 499).

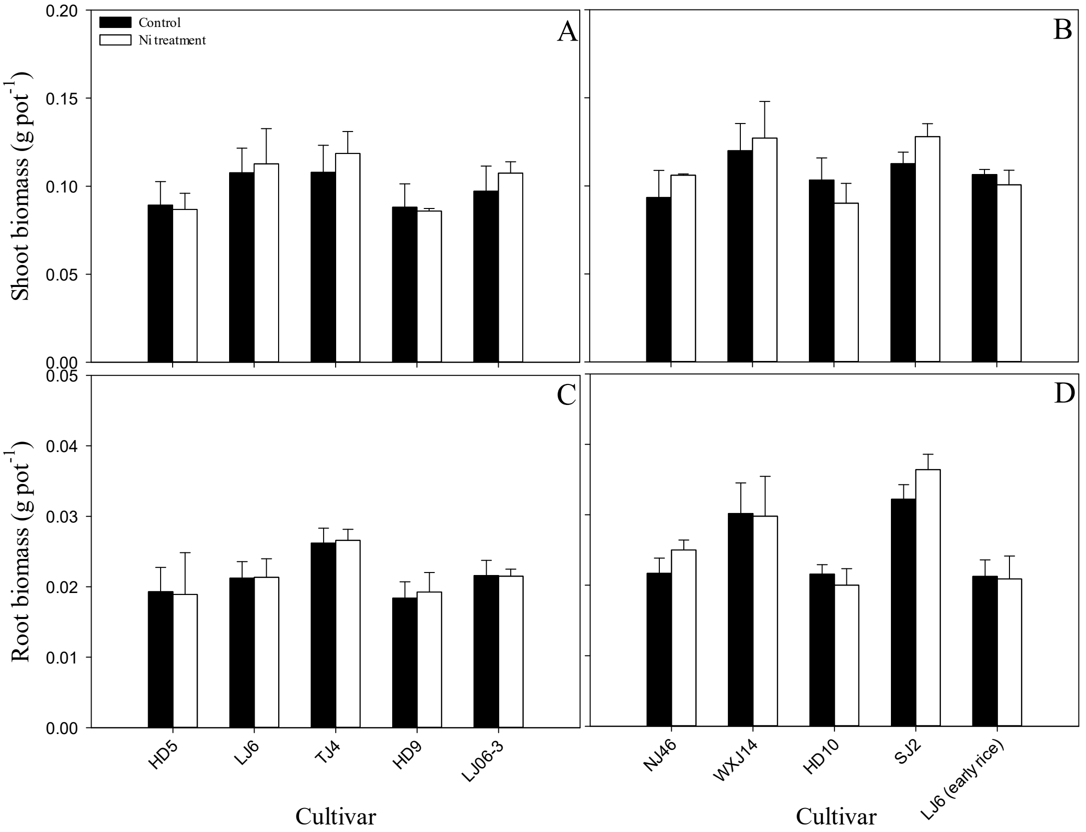

Figure S3. The biomass (dry weights) of rice seedlings after exposure to 0 and 10 μmol L^{-1} Ni for 3 days under hydroponics condition. A and C represent shoot biomass and root biomass of five lowest Ni-accumulating rice cultivars, respectively. B and D represent shoot biomass and root biomass of five highest Ni-accumulating rice cultivars, respectively. Data are means ± standard deviation (n=3).

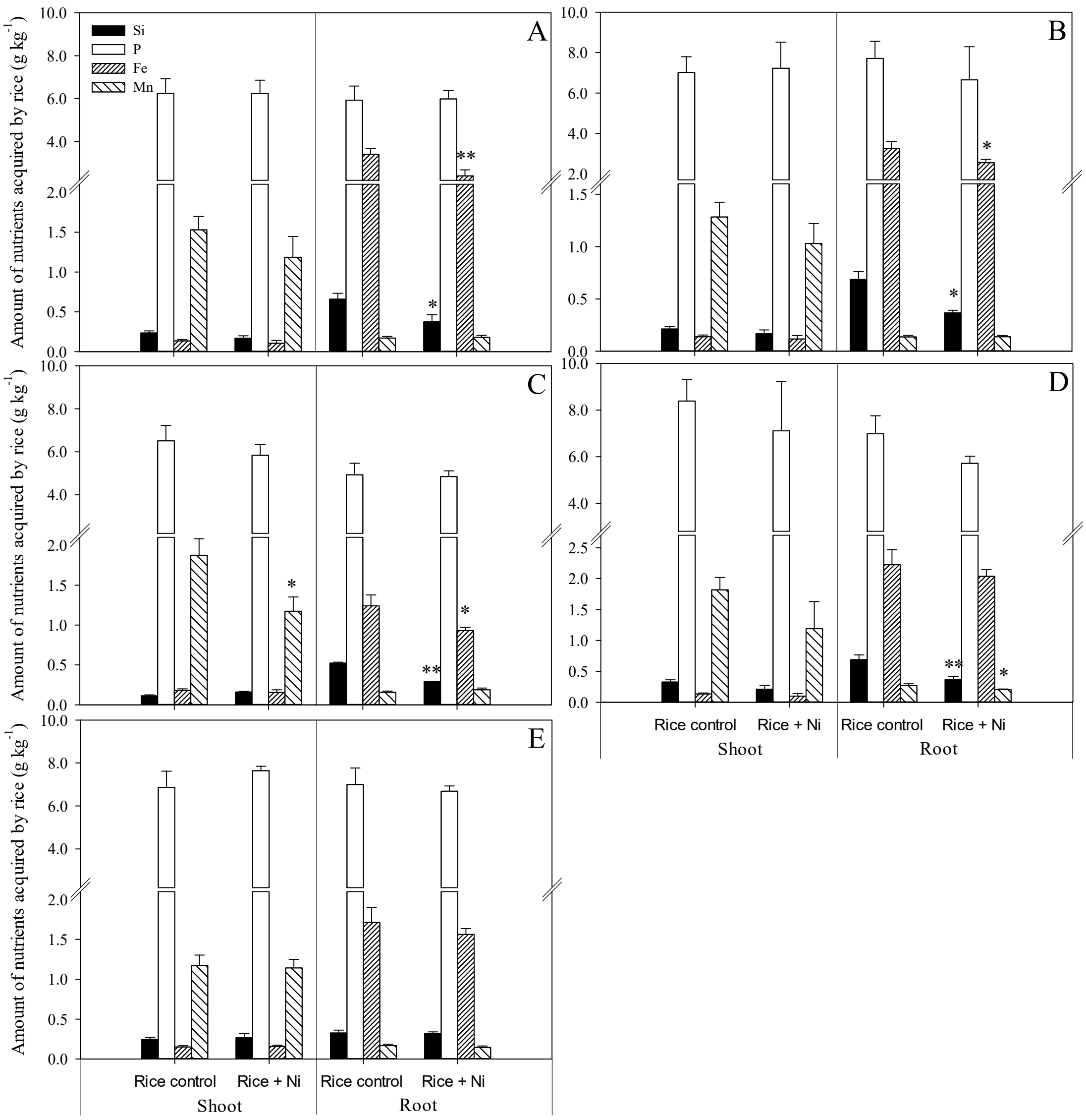

Figure S4. The amount of nutrients in rice shoots and roots with or without addition of 10 μmol L^{-1} Ni for 3 days under hydroponics condition. A, B, C, D and E represent HD5, LJ6, TJ4, HD9 and LJ06-3 (i.e., five lowest Ni-accumulating rice cultivars), respectively. Data are means ± standard deviation (n=3). *($p < 0.05$) and **($p < 0.01$) indicate a significant difference between two treatments according to an independent-samples t test.

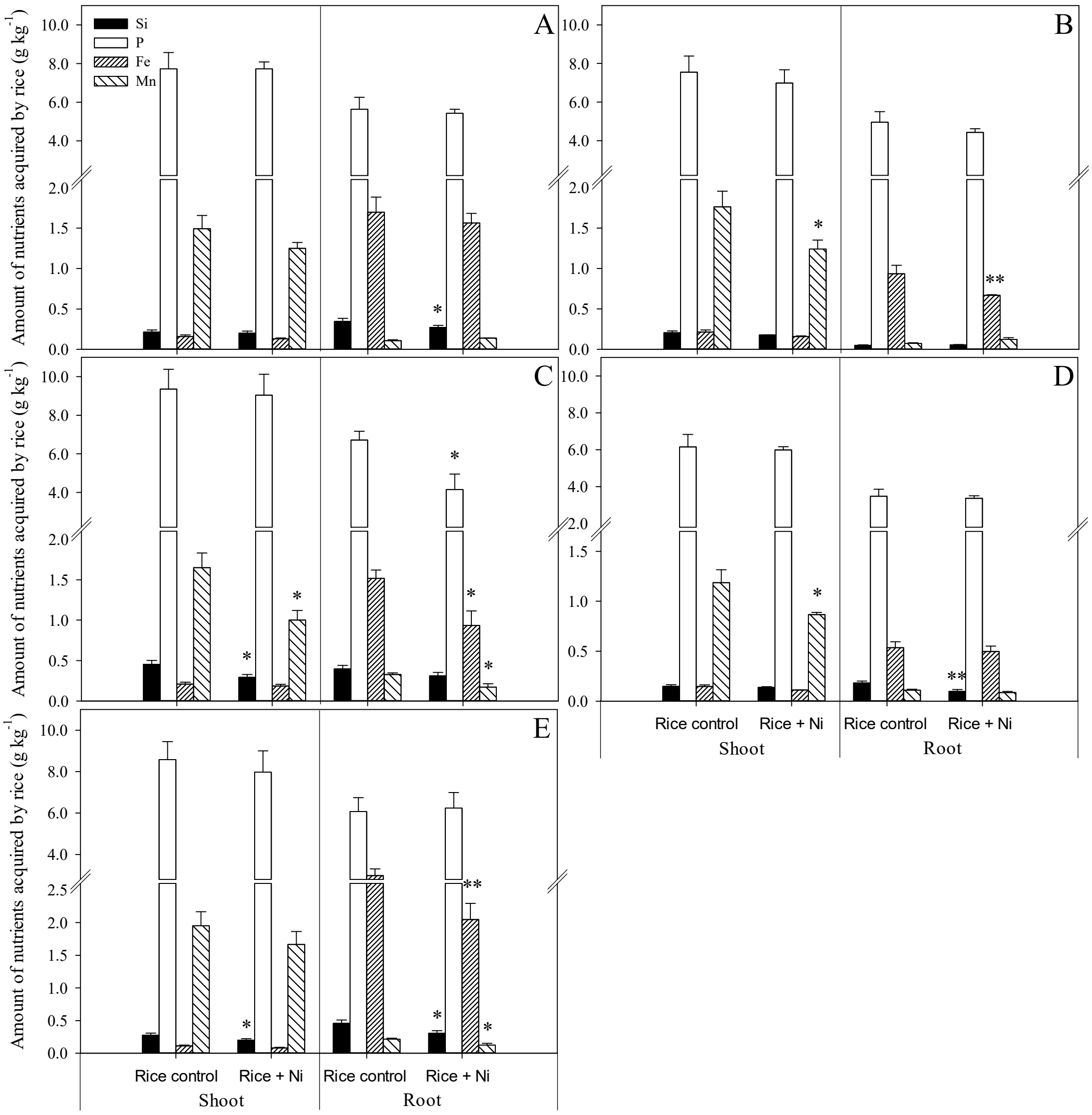

Figure S5. The amount of nutrients in rice shoots and roots with or without addition of 10 μmol L^{-1} Ni for 3 days under hydroponics condition. A, B, C, D and E represent NJ46, WXJ14, HD10, SJ2 and LJ6 (early rice) (i.e., five highest Ni-accumulating rice cultivars), respectively. Data are means ± standard deviation (n=3). *($p < 0.05$) and **($p < 0.01$) indicate a significant difference between two treatments according to an independent-samples t test.
